# Morphine and nicotine intake in high drinking in the dark mice is reduced by informatics-nominated and translationally relevant compounds

**DOI:** 10.3389/fpsyt.2026.1830225

**Published:** 2026-08-13

**Authors:** Darren E. Ginder, Jonathan Palacios, Madeleine Stewart, Bryan E. Jensen, Marissa B. Borrego, Luis Tzab, Angela R. Ozburn

**Affiliations:** 1 Department of Behavioral Neuroscience, Oregon Health and Science University, Portland, OR, United States; 2 Veterans Affairs Portland Health Care System, Portland, OR, United States

**Keywords:** high drinking in the dark mice, informatics nominated compounds, morphine, neuroinflammation, nicotine

## Abstract

**Introduction:**

Recent studies have identified an important role for neuroinflammatory signaling in alcohol and substance use. Dysregulation of neuroimmune signaling has been shown to be involved in the risk for drinking to intoxication, and pharmacologically targeting immune pathways can reduce early-stage and chronic binge-like alcohol drinking. High drinking in the dark (HDID) mice also consume other drugs of abuse, such as morphine and nicotine. Thus, this study aimed to explore anti-inflammatory compounds to reduce morphine and nicotine intake in a genetic mouse model for substance use disorder.

**Methods:**

We tested whether male and female inbred HDID mice will consume pharmacologically relevant levels of morphine and nicotine using the limited-access drinking in the dark assay (n = 7–12/sex/line). Drug intake was correlated with blood levels of morphine and nicotine metabolites (morphine-3-glucuronide and cotinine, respectively). We next tested whether the anti-inflammatory compounds terreic acid, pergolide, and apremilast would reduce morphine or nicotine intake (n = 11–14/sex/line/drug).

**Results:**

Both inbred HDID (iHDID)-1 and iHDID-2 mouse lines drank pharmacologically relevant amounts of morphine and nicotine, where we observed a significant positive correlation between morphine and morphine-3-glucuronide in iHDID-2 male mice (*p* = 0.04) and a significant positive correlation between nicotine and cotinine in iHDID-2 female mice (*p* = 0.006). Terreic acid, pergolide, and apremilast significantly reduced morphine and nicotine intake in both lines and sexes.

**Discussion:**

These findings highlight the versatility of iHDID-1 and iHDID-2 mice for both the consumption of drugs of abuse and as a model to test potential therapeutics to reduce drug intake. The results here provide further evidence that targeting inflammatory signaling offers promise for reducing opioid and nicotine use.

## Introduction

The concomitant use of nicotine in the presence of other substance use disorders (SUDs) is a public health concern, with an approximately 50% higher mortality rate in individuals diagnosed with a SUD who have concurrent nicotine use (1–3). Opioid use disorder (OUD) is of particular interest, as 2.7% of individuals 12 or older in the United States have misused opioids in the past year (4). Previous reports have shown that of those seeking treatment for OUD, 80% use cigarettes (5). Furthermore, in methadone-maintained individuals with OUD, the number of cigarettes smoked was found to be negatively correlated with treatment effectiveness (6, 7), and smoking cessation was found to aid in treatment success (6). Considering that many OUD (8–11) or nicotine (12, 13) cessation medications exhibit mixed efficacy in aiding in the cessation of using opioids and nicotine, exploration into compounds that could treat both substance use disorders simultaneously is necessary.

While there is some research into modeling polysubstance use in rats (14–16), there remains a dearth in the literature surrounding nicotine and opioid use in animal models. As such, screening a mouse model for their propensity to consume nicotine and morphine separately is important to provide the field with an animal that will volitionally consume both compounds. The inbred high drinking in the dark line 1 and line 2 (iHDID-1 and iHDID-2, respectively) mouse lines were selectively bred to reach high blood alcohol levels in the limited-access drinking in the dark (DID) assay (17, 18). HDID mice were later shown to consume higher levels of midazolam, morphine, and nicotine (in a DID assay) than their genetically heterogeneous founders (19). Humans consume nicotine and opioids orally, thus establishing an animal model of oral intake, resulting in a pharmacologically relevant amount of drug (or drug metabolites), which offers face and construct validity. Additionally, because iHDID mice have been shown to reliably drink ethanol to intoxication over a 2-day period (20, 21), iHDID mice have the benefit of being a high-throughput, early-stage model of alcohol and drug use. This high-throughput capability allows for rapid testing of compounds to aid in cessation or a decrease in drug use, where an early intervention may offer significant harm reduction. Finally, alcohol drinking and the propensity to use other drugs of abuse have been proposed to be a polygenetic trait in both mice (20, 22, 23) and humans (24, 25). Due to genetic variation, response to pharmacotherapies may differ at the individual level. Thus, an approach to compound testing that considers polygenetic contributions (i.e., testing with iHDID-1 and iHDID-2 mice) will enhance the translatability of animal testing.

Previous work with HDID used a novel bioinformatics approach to identify whether the molecular signature of risk could be used to identify novel compounds for reducing binge-like alcohol drinking (26). In Ferguson et al. (2018), we demonstrated the usefulness of the L1000 Connectivity Map (CMap) database, a high-throughput gene expression profiling resource from the NIH Library of Integrated Network-based Cellular Signatures (LINCS) program, to aid in nominating compounds to test for efficacy in reducing ethanol binge-like drinking. The database contains transcriptional signatures for thousands of compounds. We used genes that were differentially expressed in several brain regions from HDID mice and their genetically heterogeneous founders (HS/Npt mice) to query CMap LINCS, computed connectivity scores for each compound, and prioritized compounds for testing that had the opposite effects on transcription as selection for drinking to intoxication (26). We found that multiple doses of the top two scoring compounds, terreic acid and pergolide, were able to significantly reduce binge-like ethanol drinking (26). Both compounds interact with inflammatory pathways; terreic acid is a Bruton’s tyrosine kinase (BTK) inhibitor, and pergolide modulates the nucleotide-binding domain, leucine-rich-containing family, pyrin domain-containing-3 (NLRP3) inflammatory pathway by agonizing dopamine D1 receptors (27, 28). Furthermore, several genetic, animal, and human studies over the last decade have identified phosphodiesterase 4 (PDE4) as a promising target for alcohol and substance use disorders (29–37). Apremilast is a PDE4B inhibitor with anti-inflammatory actions that is FDA-approved for the treatment of plaque psoriasis, psoriatic arthritis, and Behcet’s disease (38). Notably, apremilast is effective at reducing ethanol consumption in rats (36) and mice (iHDID-1, iHDID-2, and C57BL/6J) (35). Furthermore, non-treatment-seeking individuals with Alcohol use disorder (AUD) also displayed reductions in alcohol consumption (35). These works show that apremilast is effective at reducing ethanol consumption across multiple species and across a spectrum of assays modeling different aspects of AUD, such as binge-like drinking, self-administration, vapor- or stress-facilitated drinking, and two-bottle choice using mice (35) or rats (36) and non-treatment-seeking humans (35).

In the studies reported here, we further develop the use of iHDID mice as models for nicotine and morphine use and early-stage intervention testing using the informatics-nominated compounds. Using the DID assay, iHDID-1 and iHDID-2 mice were offered either morphine or nicotine (using previously reported concentrations) (19). To test whether they consumed pharmacologically relevant amounts of drugs, we collected blood samples at the end of the DID. To account for the short half-lives of morphine and nicotine in mice, we quantified their primary metabolites. Specifically, we measured morphine-3-β-d-glucuronide (M3G) and cotinine as stable indicators of morphine and nicotine exposure, respectively. Next, as there are similar effects of ethanol, opioid, and nicotine on inflammatory response, we hypothesized that the informatics-nominated anti-inflammatory compounds (i.e., terreic acid, pergolide, and apremilast) that reduced ethanol consumption would also be effective at reducing opioid and nicotine consumption. The current pharmacological interventions for OUD and nicotine—naltrexone and varenicline, respectively—were used as positive controls.

## Methods

### Animals

Adult iHDID-1 and iHDID-2 male and female mice were bred and housed in the Veterinary Medical Unit at the Veterans Affairs Portland Health Care System (Portland, OR, USA). Mice were bred and maintained on a reverse 12-hr light/dark cycle (lights off 0730, lights on 1930 PST) with food (5LOD chow, PMI Nutrition International, St. Louis, MO, USA) and water provided *ad libitum*. Animal rooms were maintained at a temperature of 21 °C ± 1 °C. Mice were housed in standard polycarbonate cages with stainless steel wire tops on Bed-o’Cobs bedding (The Andersons Inc., Maumee, OH, USA). All mice were habituated to individual housing and sipper bottles 5–7 days prior to experimentation. All procedures were conducted in accordance with NIH Guidelines for the Care and Use of Laboratory Animals and were approved by the Portland VA Institutional Animal Care and Use Committee.

#### Morphine drinking in the dark

For the morphine experiments, two separate groups of mice were used. Animal ages at the beginning of each experiment, selection, and filial generations were as follows: Experiment 1: DID and M3G analysis (n = 7/line/sex), iHDID-1 (P182–196; S26:F30) and iHDID-2 (P182–203; S37:F20); Experiment 2: morphine DID and compound testing (n = 11–14/line/sex/treatment), iHDID-1 (P79–111; S26:F26) and iHDID-2 (P82–122; S37:F16).

#### Nicotine drinking in the dark

For the nicotine experiments, two separate groups of mice were used. Animal ages for the nicotine experiment at the beginning of each experiment, selection, and filial generations were as follows: Experiment 3: DID and cotinine analysis (n = 12/line/sex), iHDID-1 (P90–P147; S26:F30–31) and iHDID-2 (P97–P168; S26:F20–21); Experiment 4: nicotine DID and compound testing (n = 11–14/line/sex/treatment), iHDID-1 (P56-77; S26:F26) and iHDID-2 (P77–P119; S37:F16).

### Drugs

For the morphine experiments, morphine sulfate (Cayman Chemical, Ann Arbor, MI, USA) was dissolved in a 0.2% saccharin and tap water solution to a concentration of 0.7 mg/mL. For the nicotine experiments, nicotine bitartrate dihydrate (Sigma-Aldrich, St. Louis, MO, USA) was dissolved in tap water to a concentration of 50 µg/mL. Drinking solution concentrations were determined from published literature (19, 39–42).

For the compound testing portion of both experiments, all drugs were delivered via intraperitoneal (i.p.) injection. The doses selected for these studies were the lowest effective doses of terreic acid and pergolide shown to reduce binge-like drinking without altering water or saccharin intake or locomotor activity (19). Similarly, the dose for apremilast was chosen based on previously published data showing efficacy of reducing ethanol intake (35, 43) while balancing reductions in water/saccharin intake (35). Terreic acid was dissolved in sterile saline and administered at a dose of 7.5 mg/kg (26). Pergolide was dissolved in a 1% dimethyl sulfoxide (DMSO)/saline solution and administered at a dose of 2 mg/kg (26). Apremilast was suspended in a 1.75% Tween-20/saline solution and administered at a dose of 40 mg/kg (35). For the morphine experiment, naltrexone was dissolved in sterile saline and administered at a dose of 1 mg/kg (17). Finally, for the nicotine experiment, varenicline was dissolved in sterile saline and administered at a dose of 3 mg/kg (44).

### Drinking in the dark

**Figure 1:**
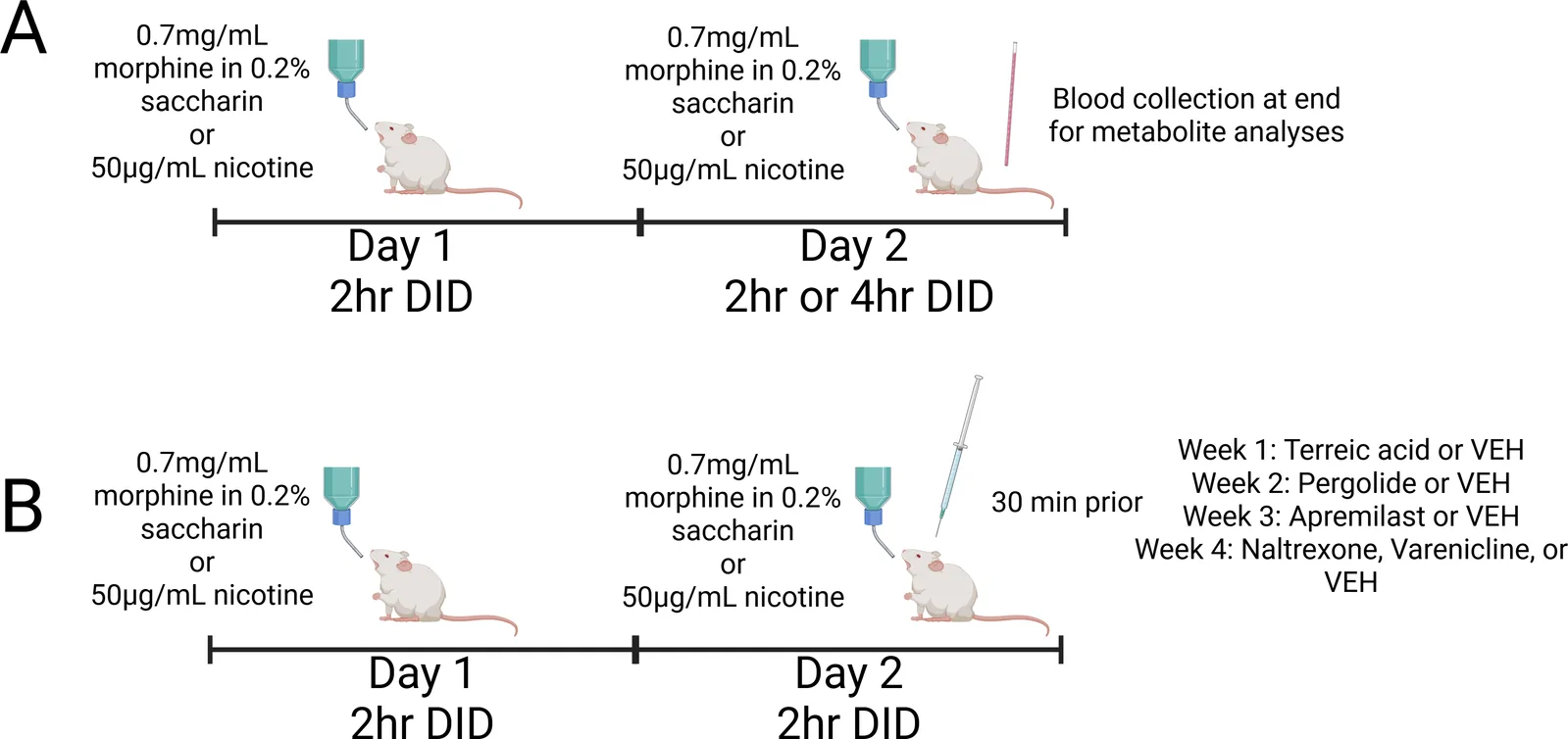
Experimental designs and timelines. In Experiment 1 **(A)**, a 2-day drinking in the dark (DID) assay was used to determine if inbred high DID (iHDID)-1 and iHDID-2 mice would drink a morphine or nicotine solution. Blood was collected after the DID assay to assess the morphine metabolite morphine-3-β-d-glucuronide (M3G) (4 hr) or the nicotine metabolite cotinine (2 hr). Similar to Experiment 1, Experiment 2 **(B)** used a 2-day, 2-hr DID assay to determine the effectiveness of treatment compounds (pergolide 2 mg/kg, terreic acid 7.5 mg/kg, and apremilast 40 mg/kg) on reducing morphine or nicotine consumption. Naltrexone (1 mg/kg) and varenicline (3 mg/kg) were used as positive controls. Each DID assay and corresponding treatment were separated by 1 week. Created with Biorender.com.

A modified 2-day DID assay (20) was used to test morphine and nicotine consumption in iHDID-1 and iHDID-2 mice. HDID mice were previously shown to reach high blood ethanol concentrations with a 2-day DID assay (18, 20), and we have shown that a 2-day DID is sufficient for testing immunosuppressants for ethanol intake (21); therefore, we employed a 2-day DID to assess morphine consumption (see **Figure 1A** for timeline). To assess nicotine consumption, a similar modified DID protocol as the morphine DID was followed, with the only change being a 2-hr access period on day 2 for nicotine. The 2-hr access period was deemed appropriate due to the LD_50_ of nicotine in mice (3.3 mg/kg) (45) and, based on our previous work, day 2 had the highest nicotine intake (19) (see **Figure 1A** for timeline).

#### Morphine drinking in the dark

The morphine DID followed a similar protocol to previous publications (19, 20): on day 1, mice were provided with a 2-hr access period to 0.7 mg/mL morphine/saccharine drinking solution. Volume measurements were recorded at the start (0 hr) and at the end (2 hr) of the access period. On day 2, the access period was extended to 4 hr, with volume recordings occurring at the start (0 hr), mid-point (2 hr), and end (4 hr) of the access period. Differences in drinking tube volume between timepoints on days 1 and 2 were used to calculate the morphine dose achieved. Blood was collected after the 4-hr period on day 2 for M3G analyses. Correlations between M3G plasma concentration and morphine intake were performed on DID data from the 2–4-hr timepoint on day 2 with M3G concentration.

#### Morphine-3-glucuronide concentration

To determine whether a pharmacologically relevant dose of morphine was consumed during the DID assay, blood was collected and analyzed for the active metabolite M3G, which has been shown to be a valid biomarker for morphine (46) and has a half-life of 27 min (47). At the end of the second day of the DID assay, 50 µL of blood was collected from the retro-orbital sinus and placed in microtubes containing 5 µL of 3.2% sodium citrate. Plasma was then extracted from blood samples by centrifugation at 2,000 *g* for 10 min at room temperature. Approximately 10 µL of plasma was then collected from each sample and stored at −80 °C. Plasma was then packaged on dry ice and sent to the Oregon Health & Science Bioanalytical Shared Resource/Pharmacokinetics Core for M3G analysis.

#### Chemicals and reagents

M3G and internal standard morphine-d3-3-β-d-glucuronide (d3-M3G) solutions were purchased from Cerilliant Corporation (Round Rock, TX, USA). Solvents, including water and methanol for liquid chromatography–tandem mass spectrometry (LC-MS/MS) analysis and sample preparation, were from VWR (Tualatin, OR, USA). Sodium Citrate CD1 mouse plasma was obtained from Innovative Research (Novi, MI, USA). Silanized 13 × 100 glass tubes were obtained from Thermo Fisher Scientific (Waltham, MA, USA), and silanized inserts were obtained from SunSri (Rockwood, TN, USA).

#### Preparation of mouse plasma calibrator sample and unknown samples

Commercial stock solutions of M3G (1:1 in methanol:water) and d3-M3G in methanol with 0.05% sodium hydroxide were stored at 4 °C. Internal standard crash/extraction solution was prepared fresh for each batch at 0.1 ng/µL in 1:1 acetonitrile:methanol solution and kept on ice.

Calibration curve was prepared by spiking stock solutions into drug-free Sodium Citrate CD1 mouse plasma and performing subsequent serial dilutions to attain 10 to 10,000 ng/mL final calibrator concentrations.

Ten microliters of calibrators or unknown samples was crashed by adding 50 μL of internal standard crash/extraction solution. Samples were vortexed on the pulsing vortex setting for 2 min at 2,500 rpm. Samples were then centrifuged at a maximum of 13,500 rpm for 2 min. Supernatant was removed to silanized 13 × 100 glass tubes and dried in a Biotage TurboVap (Uppsala, Sweden) for 5 min at 1 L/min flow; the water bath was maintained at 40 °C. Samples were then reconstituted in 50 µL of 38:2:0.04 water:methanol:formic acid and transferred to silanized inserts. Ten microliters of the final mixture was injected for analysis.

#### High-performance liquid chromatography–tandem mass spectrometry analysis

The M3G and d3-M3G were analyzed using a Sciex 4000 QTRAP hybrid/triple quadrupole linear ion trap mass spectrometer (Foster City, CA, USA) with electrospray ionization in the positive mode. The mass spectrometer was interfaced to a Shimadzu HPLC system (Columbia, MD, USA) with SIL-20AC XR auto-sampler, LC-20AD XR LC pumps, and CTO-20AC column oven. The mass spectrometer was operated with the following settings: source voltage 5,500 V, GS1 40, GS2 50, CUR 10, TEM 700, and CAD gas high.

M3G was quantified with multiple reaction monitoring (MRM), and the MS/MS transitions used were optimized by infusion of pure compound solutions with method settings as presented in **Table 1**. The bold transition was used for quantification, with alternate transitions used for peak qualification to ensure method specificity (quantifier and qualifier peak ratio in unknown samples was within ±20% of the mean quantifier and qualifier peak ratio in the calibration curve reference standard). M3G was separated from method interferents using a Restek Corporation Raptor Biphenyl column 50 × 2.1 (Bellefonte, PA, USA) kept at 40 °C using a column oven. The gradient mobile phase was delivered at a flow rate of 0.5 mL/min and consisted of two solvents: (A) 2 mM ammonium formate and 0.1% formic acid in water and (B) 2 mM ammonium formate and 0.1% formic acid in methanol. The initial concentration of solvent B was 5%, held for 0.1 min, followed by a linear increase to 40% B by 1.5 min, then to 95% B in 3.7 min, then to 98% over 1.8 min, decreased back to the starting 5% B over 0.1 min, and then held for 0.4 min. LC-MS/MS data were acquired using Analyst 1.6.2 and processed using the MultiQuant 3.0.3 software.

**Table 1:** Tandem mass spectrometry (MS/MS) settings.

Parent mass (*m*/*z*)	Product mass (*m*/*z*)	MS/MS Scan dwell time (msec)	MS/MS Parent to product ion transition monitored	DP voltage	EP voltage	CE voltage	CXP voltage
462.2	286.1	50	M3G Quantifying transition	20	10	40	10
462.1	201.1	50	M3G Qualifying transition	60	10	60	5
465	289.1	50	d3-M3G	40	10	40	10

CE, collision energy; CXP, collision cell exit potential; DP, Declustering potential; EP, Entrance potential; M3G, morphine-3-β-d-glucuronide; d3-M3G, morphine-d3-3-β-d-glucuronide.

#### Method performance

The validated linear analytical measurement range was 10–5,000 ng/mL. The lower limit of quantification (LLOQ) for M3G was 10 ng/mL with a between-run (n = 3) accuracy of 94% and precision Relative standard deviation (RSD) of 6.5% (accuracy for all other calibrators was within ±20%, and precision was <20%). Recovery for 100 ng/mL M3G spiked into matrix sample was approximately 92%. Mouse plasma stability QC sample spiked at 100 ng/mL and stored frozen at −70°C underwent degradation of 6% over 23 days. This method was modified from a previously published protocol (48).

#### Nicotine drinking in the dark

Day 1 followed the same procedure as the morphine DID; mice were provided access to sipper tubes containing 50 µg/mL of nicotine, and volume measurements were recorded at the start (0 hr) and end (2 hr) of the access period. Day 2 followed the same procedure: a 2-hr DID with volume measurements recorded at the start (0 hr) and end (2 hr) of the access period. Blood was collected at the end of the access period for blood cotinine analyses.

#### Cotinine concentration

To determine whether a pharmacologically relevant dose of nicotine was consumed during the DID assay, blood was collected and analyzed for the nicotine metabolite, cotinine. The half-life of nicotine in mice is 6–7 min (49, 50), whereas the half-life of cotinine is 30–50 min (50). At the end of the second day of the DID assay, 50 μL of blood was collected from the retro-orbital sinus, allowed to clot at room temperature for 30 min, and then centrifuged at 1,300 *g* for 10 min to separate serum from blood. Serum was collected, and cotinine levels were measured via enzyme-linked immunosorbent assay (ELISA; Calbiotech Inc., El Cajon, CA, USA). Absorbance (A_450_) measurements were obtained on a BioTek Epoch 2 (BioTek, Winooski, VT, USA) microplate spectrophotometer. A standard curve was created by calculating the reciprocal of the absorbance measure (1/A_450_) of each standard. Sample cotinine levels were determined by fitting absorbance values to the standard curve.

### Drinking in the dark and serial compound testing

#### Morphine

A 4-week serial DID was used to assess the efficacy of terreic acid, pergolide, and apremilast, with naltrexone used as a positive control. A similar DID protocol was followed as previously published (20), with adherence to a 2-hr DID for both day 1 and day 2; a 2-day DID assay with 10-mL sipper tubes filled with a morphine solution (0.7 mg/mL) and volume measurements occurred at the start (0 hr) and end (2 hr) of the access period for days 1 and 2. On day 1 of the DID procedure, mice were provided with 2-hr access to the morphine solution. On day 2 of the DID procedure, mice were injected with either vehicle or one of the treatment compounds 30 min prior to access to sipper tubes containing the morphine solution. Only one treatment compound was administered per week, with a different compound tested each week across the 4-week period (i.e., week 1, terreic acid or vehicle; week 2, pergolide or vehicle; week 3, apremilast or vehicle; and week 4, naltrexone or vehicle—see **Figure 1B** for the experimental timeline). Mice were pseudo-randomly assigned to new treatment groups each week, ensuring that day 1 intake was similar across groups prior to compound testing on day 2. Groups were balanced based on sex and strain.

#### Nicotine

**Figure 2:**
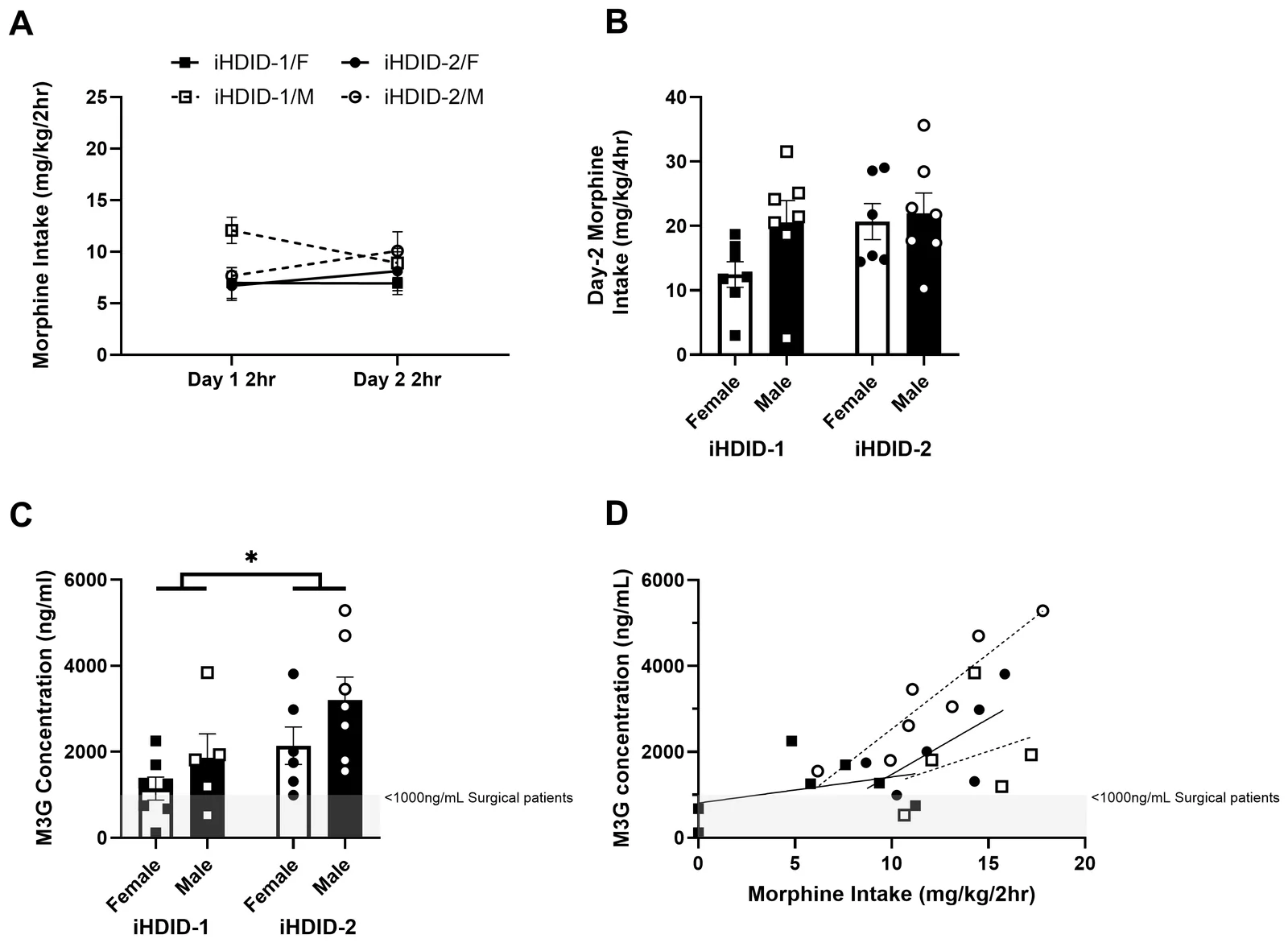
Inbred high drinking in the dark (iHDID)-1 and iHDID-2 mice drink pharmacologically relevant doses of morphine. Average intake of iHDID-1 and iHDID-2 mice over the 2-day drinking in the dark (DID) assay **(A)**. No differences based on sex or line were found on day 2 of the DID procedure, suggesting that all mice drank similar amounts **(B)**. iHDID-2 mice reached significantly higher plasma concentrations of morphine-3-β-d-glucuronide (M3G) than iHDID-1 mice **(C)**. Morphine intake and plasma M3G concentrations had medium-to-strong correlations for all mice except iHDID-1 males, with iHDID-2 males displaying a significant correlation between intake and M3G concentration **(D)**. *p < 0.05.

Similar to the morphine DID, a 4-week serial DID was used to assess the efficacy of terreic acid, pergolide, and apremilast; varenicline was used as a positive control. The serial DID assay followed a similar protocol to the nicotine DID: a 2-day DID assay with 10-mL sipper tubes filled with a nicotine solution (50 µg/mL) and measured at the start (0 hr) and end (2 hr) of the procedure. On the first day of the DID procedure, mice were provided with 2-hr access to the nicotine solution. On the second day, mice were injected with either vehicle or treatment compound 30 min prior to access to sipper tubes containing nicotine solution. This schedule was repeated for the duration of the experiment (4 weeks total) with a different compound tested each week (i.e., week 1, mice were administered terreic acid or vehicle; week 2, mice were administered pergolide or vehicle; week 3, mice were administered apremilast or vehicle; and week 4, mice were administered varenicline or vehicle—see **Figure 2B** for experimental timeline). Mice were pseudo-randomly assigned to new treatment groups each week based on intake. Groups were balanced based on sex and strain.

### Statistics

All statistical analyses were performed in the GraphPad Prism software v11 (Boston, MA, USA).

#### Morphine drinking in the dark

Morphine intake was analyzed using a repeated measures three-way analysis of variance (ANOVA) with day as the within-subjects variable, and sex and line as the between-subjects variables. As the day 2 morphine dose was used for correlational analyses with M3G concentration, a two-way ANOVA was used to determine differences in total (0–4 hr) day 2 morphine intake with sex and line as variables. Plasma M3G concentration was analyzed using a two-way ANOVA with sex and line as variables. Finally, a Pearson’s correlation was conducted on plasma M3G concentration and day 2, 2–4-hr timepoint morphine intake.

#### Morphine drinking in the dark and serial compound testing

Analyses were performed on day 2 intake data on each mouse line separately. Similar to the morphine DID experiment, day 2 intake at 0–2-hr and 2–4-hr timepoints was averaged for analyses. Two-way ANOVA revealed no main effect of sex or an interaction between sex and treatment; therefore, morphine intake was analyzed using a Student’s *t*-test, with dose as a variable; sex was collapsed for all groups.

#### Nicotine drinking in the dark

Nicotine intake was analyzed using a repeated measures three-way ANOVA with day as the within-subjects variable, and sex and line as between-subjects variables. As day 2 nicotine intake was used for correlational analyses, a two-way ANOVA was used to investigate differences in day 2 nicotine intake with sex and line as variables. Blood cotinine levels were analyzed using a two-way ANOVA with sex and line as variables. Finally, a Pearson’s correlation was performed on blood cotinine levels and day 2 nicotine intake.

#### Nicotine drinking in the dark and serial compound testing

Analyses were performed on day 2 intake data and on each mouse line separately. Two-way ANOVA revealed no main effect of or interaction with sex; therefore, nicotine intake was analyzed using a Student’s *t*-test, with treatment as a variable; sex was collapsed for all groups.

## Results

### Morphine drinking in the dark

#### Morphine intake

A repeated measures three-way ANOVA revealed a significant main effect of sex [*F*(1,23) = 4.573, *p* = 0.043] and a significant day × line interaction [*F*(1,23) = 4.515, *p* = 0.045], but no impact of day [*F*(1,23) = 0.035, *p* = 0.854] or line [*F*(1,23) = 0.247, *p* = 0.624] and no other significant interactions: day × sex [*F*(1,23) = 0.412, *p* = 0.527], line × sex [*F*(1,23) = 0.789, *p* = 0.384], and day × line × sex [*F*(1,23) = 1.569, *p* = 0.223] over the 2-day DID period. Post-hoc analyses of the day × line interaction revealed no significant comparisons between lines [*t*(46) < 3.00, *p* > 0.05] (**Figure 2A**), suggesting that male mice of both strains consumed more morphine than females of both strains, and that the differences based on sex and day are dependent upon the intake of males of both lines.

#### Day 2 morphine intake

A two-way ANOVA on day 2 morphine intake revealed no significant differences between iHDID-1 and iHDID-2 mice based on sex [*F*(1,23) = 2.656, *p* = 0.12], line [*F*(1,23) = 2.789, *p* = 0.11], and no sex by line interaction [*F*(1,23) = 1.372, *p* = 0.25] (**Figure 2B**). This suggests that both lines and sexes consumed similar amounts of morphine during day 2 of the DID procedure.

#### Morphine-3-glucuronide analysis

Analyses of plasma M3G concentration revealed a significant effect of line [*F*(1,21) = 6.663, *p* = 0.017] but no effect of sex [*F*(1,21) = 3.859, *p* = 0.063] and no sex × line interaction [*F*(1,21) = 0.150, *p* = 0.703], indicating that iHDID-2 mice, both males and females, achieved higher M3G concentrations compared to iHDID-1 mice (**Figure 2C**). Correlational analyses between M3G plasma concentration and morphine intake from hours 2–4 revealed a significant correlation in iHDID-2 male mice [*r*(5) = 0.927, *p* = 0.00270] but not in iHDID-1 males [*r*(3) = 0.321, *p* = 0.599], iHDID-1 females [*r*(5) = 0.374, *p* = 0.409], or iHDID-2 females [*r*(4) = 0.675, *p* = 0.141] (**Figure 2D**). These results show that plasma M3G concentration levels are correlated with morphine intake in iHDID-2 male mice.

### Morphine drinking in the dark and serial compound testing

#### iHDID-1 serial drinking in the dark

**Figure 3:**
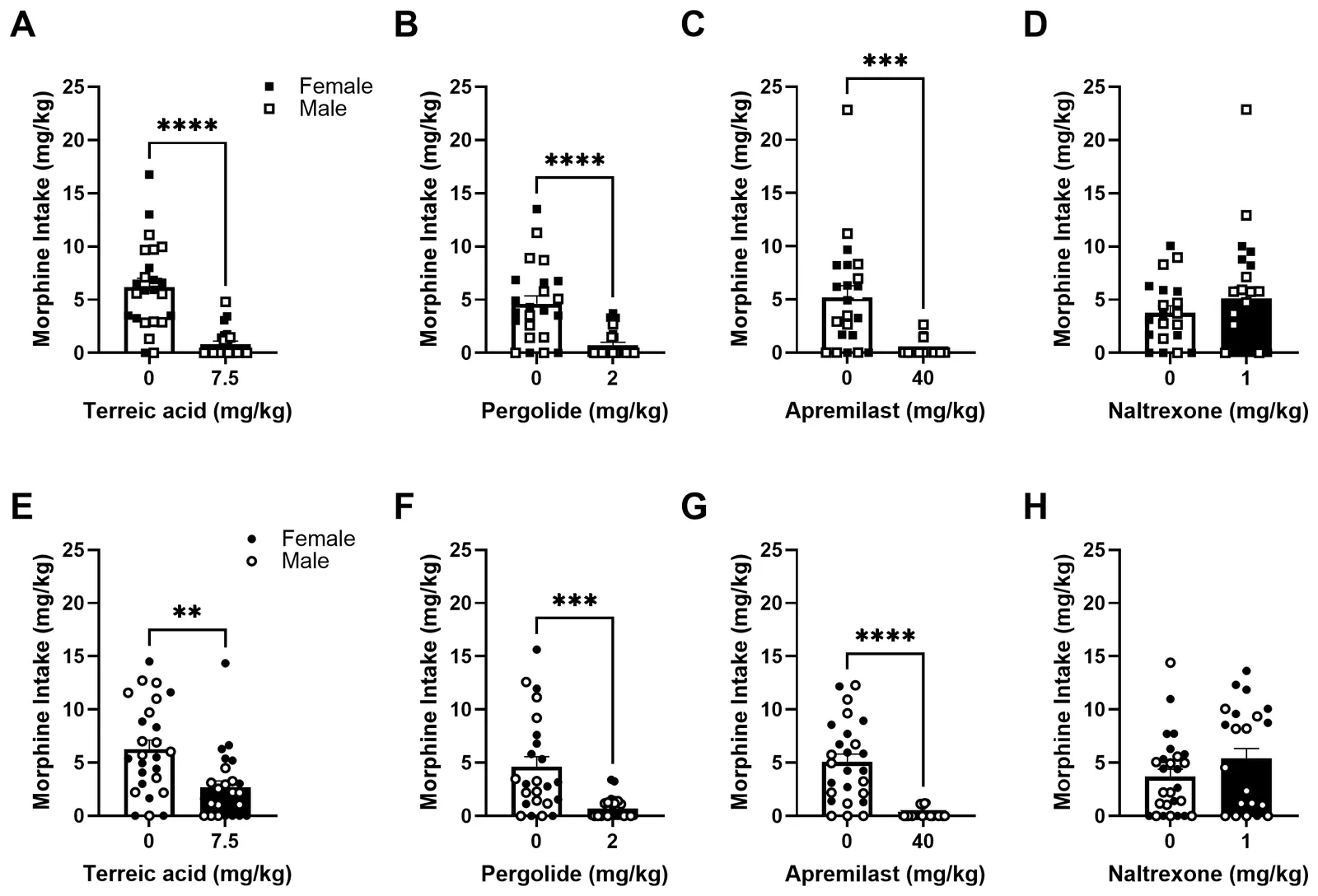
Terreic acid, pergolide, and apremilast reduced morphine intake in both inbred high drinking in the dark (iHDID)-1 and iHDID-2 mice. iHDID-1 morphine consumption was reduced with treatment. Terreic acid **(A)**, pergolide **(B)**, and apremilast **(C)** all significantly reduced morphine consumption on day 2 of the DID procedure compared to vehicle (VEH)-treated mice. Naltrexone **(D)** was used as a positive control and did not impact morphine consumption on day 2. iHDID-2 mice followed a similar outcome, with terreic acid **(E)**, pergolide **(F)**, and apremilast **(G)** reducing morphine intake, while naltrexone **(H)** did not alter morphine intake. *p < 0.05, **p < 0.001, ***p = 0.0001, and ****p < 0.0001.

Analyses of iHDID-1 mice during the serial DID procedure revealed a significant effect from all informatics-nominated compounds. Terreic acid significantly reduced morphine intake [*t*(23) = 5.565, *p* = 0.00001] (**Figure 3A**). Pergolide significantly reduced morphine intake [*t*(22) = 4.976, *p* = 0.000046] (**Figure 3B**). Apremilast significantly reduced morphine intake [*t*(22) = 4.789, *p* = 0.00012] (**Figure 3C**). Interestingly, the current pharmacological intervention, naltrexone [*t*(19) = 0.752, *p* = 0.29], did not alter morphine intake (**Figure 3D**). Together, these data highlight that informatics-nominated compounds are effective at reducing morphine intake in iHDID-1 mice but not the current pharmacological intervention (naltrexone).

#### iHDID-2 serial drinking in the dark

Analyses of iHDID-2 mice during the serial DID procedure revealed a significant effect from all informatics-nominated compounds. Terreic acid significantly reduced morphine intake [*t*(25) = 3.489, *p* = 0.0011] (**Figure 3E**). Pergolide significantly reduced morphine intake [*t*(23) = 4.215, *p* = 0003] (**Figure 3F**). Apremilast significantly reduced morphine intake [*t*(25) = 6.784, *p* = 0.00000052] (**Figure 3G**). Similar to iHDID-1 mice, naltrexone had no effect on morphine intake [*t*(24) = 1.713, *p* = 0.14] (**Figure 3H**). These data indicate that informatics-nominated compounds are effective at reducing morphine intake in iHDID-2 mice but not the current pharmacological intervention (naltrexone).

### Nicotine drinking in the dark

#### Nicotine intake

**Figure 4:**
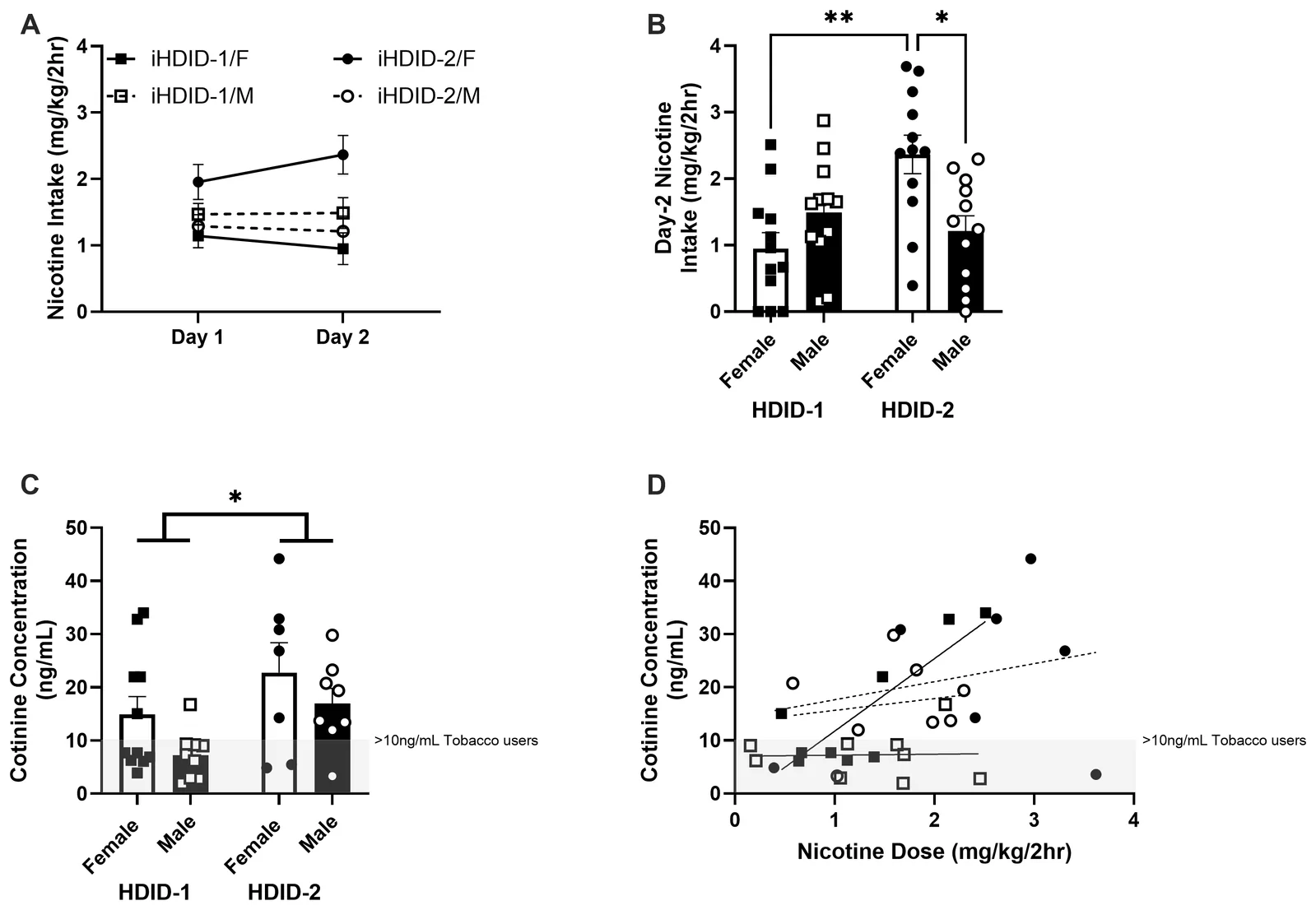
Inbred high drinking in the dark (iHDID)-1 and iHDID-2 mice drink pharmacologically relevant doses of nicotine. Average intake of iHDID-1 and iHDID-2 mice over the 2-day drinking in the dark (DID) assay **(A)**. Female iHDID-2 consumed more nicotine compared to male iHDID-2 and female iHDID-1 on day 2 of the DID assay **(B)**. iHDID-2 mice reached significantly higher concentrations of cotinine than iHDID-1 mice **(C)**. Nicotine intake and serum cotinine concentrations had medium-to-strong correlations for all mice except iHDID-1 males **(D)**. *p < 0.05; **p < 0.001.

A repeated measures three-way ANOVA revealed no impact of sex [*F*(1,44) = 1.619, *p* = 0.21], or day [*F*(1,44) = 0.101, *p* = 0.752], but a significant effect of line [*F*(1,44) = 5.646, *p* = 0.022], and a significant sex × line interaction [*F*(1,44) = 12.87, *p* = 0.0008]. Female iHDID-2 mice drank significantly more nicotine solution compared to both iHDID-1 females [*t*(88) = 4.45, *p* = 0.0007] and iHDID-2 males [*t*(88) = 3.62, *p* = 0.013]. No other sex × line interactions reached significance [*t* < 2.500, *p* > 0.05]. All other interactions were non-significant: day × line [*F*(1,44) = 1.023, *p* = 0.317], day × sex [*F*(1,44) = 0.304, *p* = 0.584], or a day × sex × line [*F*(1,44) = 1.926, *p* = 0.172]. These results show that female iHDID-2 mice drank more nicotine solution as compared to males of either line or female iHDID-1 mice (**Figure 4A**).

#### Day 2 nicotine intake

Analyses of day 2 nicotine intake uncovered a significant effect of line [*F*(1,44) = 5.245, *p* = 0.027] and a sex × line interaction [*F*(1,44) = 11.48, *p* = 0.0015] but no effect of sex [*F*(1,44) = 1.517, *p* = 0.225]. *Post-hoc* analyses indicated that female iHDID-2 mice drank more nicotine solution than female iHDID-1 [*t*(44) = 4.015, *p* = 0.0014] and male iHDID-2 [*t*(44) = 3.267, *p* = 0.013] but not male iHDID-1 mice [*t*(40) = 2.490, *p* = 0.096]. All other *post-hoc* analyses were non-significant [*t*(40) < 1.000, *p* > 0.05] (**Figure 4B**).

#### Cotinine concentration

Analyses indicated that blood cotinine levels were significantly different by line [*F*(1,31) = 6.336, *p* = 0.017], revealing that iHDID-2 mice had higher concentrations of cotinine in their blood compared to iHDID-1 mice. There was no impact of sex [*F*(1,31) = 3.744, *p* = 0.062], and no sex × line interaction was present [*F*(1,31) = 0.0711, *p* = 0.792] (**Figure 4C**). Finally, correlational analyses indicated a significant correlation between blood cotinine levels and nicotine intake (mg/kg/2 hr) in iHDID-2 females [*r*(5) = 0.83, *p* = 0.006] but not in iHDID-1 males [*r*(7) = 0.033, *p* = 0.93], iHDID-1 females [*r*(7) = 0.60, *p* = 0.24], or iHDID-2 males [*r*(6) = 0.70, *p* = 0.16] (**Figure 4D**). These results show that blood cotinine levels are correlated with nicotine intake in female iHDID-2 mice, which had the highest intake and highest blood cotinine levels.

### Nicotine drinking in the dark and serial compound testing

#### iHDID-1 serial drinking in the dark

**Figure 5:**
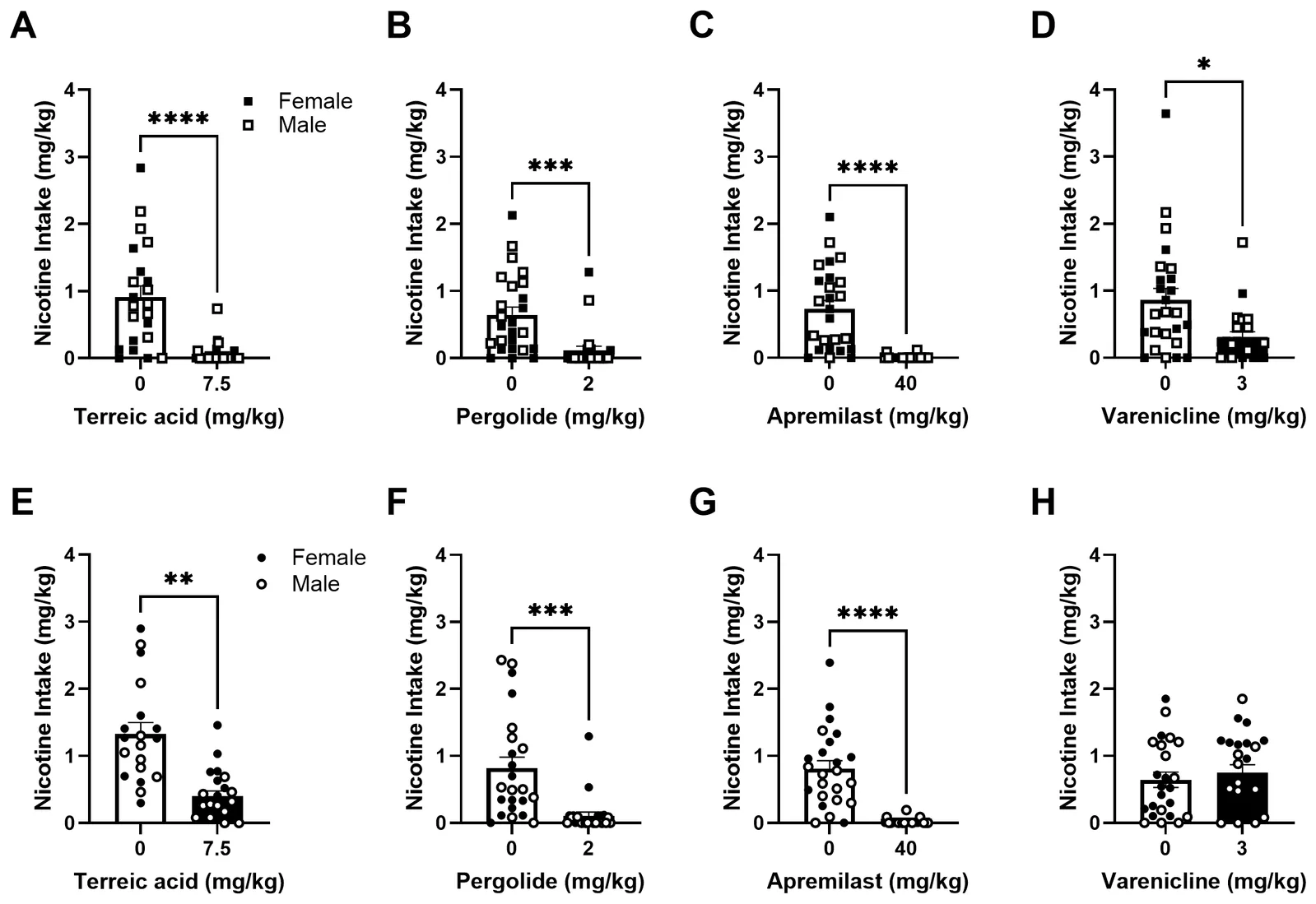
Terreic acid, pergolide, apremilast, and varenicline reduced nicotine intake in inbred high drinking in the dark (iHDID)-1 and iHDID-2 mice. iHDID-1 nicotine consumption was reduced with treatment. Terreic acid **(A)**, pergolide **(B)**, and apremilast **(C)** all significantly reduced nicotine consumption on day 2 of the DID procedure compared to vehicle (VEH)-treated mice. Varenicline **(D)** was used as a positive control and resulted in reduced nicotine consumption on day 2. Similarly, iHDID-2 mice exhibited reduced nicotine consumption with terreic acid **(E)**, pergolide **(F)**, and apremilast **(G)**. Interestingly, varenicline **(H)** did not impact nicotine intake. *p < 0.05, **p < 0.001, ***p = 0.0001, and ****p < 0.0001.

Analyses of iHDID-1 mice during the serial DID procedure revealed a significant effect of all compounds. Terreic acid treatment significantly reduced nicotine intake [*t*(21) = 5.318, *p* = 0.000047] (**Figure 5A**). Pergolide significantly reduced nicotine intake [*t*(23) = 3.817, *p* = 0.00029] (**Figure 5B**). Apremilast significantly reduced nicotine intake [*t*(24) = 5.866, *p* = 0.000004] (**Figure 5C**). Finally, varenicline also reduced nicotine intake [*t*(23) = 2.509, *p* = 0.0050] (**Figure 5D**). Together, these data show that iHDID-1 mice significantly reduced their DID nicotine intake in response to informatics-nominated compounds and the current pharmacological intervention.

#### iHDID-2 serial drinking in the dark

Analyses of iHDID-2 mice during the serial DID procedure revealed a significant effect of all compounds except varenicline. Terreic acid significantly reduced nicotine intake [*t*(15) = 3.642, *p* = 0.000044] (**Figure 5E**). Pergolide significantly reduced nicotine intake [*t*(22) = 4.059, *p* = 0.00031] (**Figure 5F**). Apremilast significantly reduced nicotine intake [*t*(23) = 6.456, *p* = 0.00000086] (**Figure 5G**). Interestingly, varenicline did not reduce nicotine intake [*t*(23) = 0.659, *p* = 0.50] (**Figure 5H**). Together, these data show that iHDID-2 mice significantly reduced their DID nicotine intake in response to informatics-nominated compounds, but not the current pharmacological intervention (varenicline).

## Discussion

Here, we report that the iHDID mouse lines selectively bred for binge-like ethanol consumption will also consume morphine and nicotine to pharmacologically relevant levels. Furthermore, the active metabolites of these two compounds (M3G and cotinine) were positively correlated with intake, highlighting that these mice will consume a measurable amount of each drug. Both M3G and cotinine have been shown to be reliable biomarkers for morphine and nicotine use, respectively (46, 51, 52). The concentration of M3G in plasma reported here was found to be similar to (53, 54), or greater than (55–57), that found in many human studies (see **Figures 2C, D**). While these human studies have a wide range of plasma M3G levels, all the authors report that the doses of morphine were therapeutically relevant (53–57); therefore, our reported plasma concentration of M3G is a valid measure of morphine intoxication. The strain differences found in M3G concentration but not in morphine intake may be a function of differences in consummatory behavior or metabolism between strains. Previous studies revealed line differences in consummatory behavior for ethanol, with HDID-1 mice displaying larger drinking bout size (“gulpers”), while HDID-2 mice exhibited increased number of drinking bouts and lower inter-bout intervals (“sippers”) (58). Future work could evaluate whether this holds true for other drugs of abuse where the use of lickometers offers intake data closer to the blood sample collection. Moreover, while there are no strain differences in ethanol metabolism (59), clearance studies would offer insight into whether there are line differences in drug metabolism. Additionally, ongoing work is underway to better understand genetic differences between iHDID-1, iHDID-2, and HS/Npt mice, which will illuminate potential genetic contributions.

While gastric absorption of morphine is typical in humans (many medications are consumed orally), the typical route of absorption for nicotine in humans is not via the gastrointestinal tract. Nevertheless, nicotine has been shown to be absorbed by the gastrointestinal tract in humans (60). Pairing our data with the work from Ivey et al. (1978) suggests that, while our route of administration exposes nicotine to first-pass metabolism, gastric absorption of nicotine is still a valid route of administration. Furthermore, cotinine will pass the blood–brain barrier (61, 62) and is psychoactive, interacting with acetylcholine receptors (62). Next, we report here that both sexes of iHDID-1 and iHDID-2 mice reached serum cotinine levels above 10 ng/mL, which is similar to daily human smokers (51, 63, 64) (see **Figures 4C, D**) and a vapor exposure model in rats (65). Furthermore, we report that iHDID-1 and iHDID-2 mice maintained their nicotine intake. As nicotine consumption can often lead to gastric discomfort (60) and its flavor can result in aversive behavior (66), our data showing that these mouse strains continued to consume nicotine following initial exposure suggest that the rewarding properties of nicotine outweigh any aversive properties. Therefore, we suggest that our model of oral consumption is a valid means of drug administration for morphine and nicotine. While we have not yet tested for behavioral outcomes with either morphine or nicotine, this is a planned next step, as there are expected behavioral outcomes of consumption of both drugs.

Next, we report that multiple informatics-nominated compounds that were effective at reducing ethanol consumption in iHDID-1 and iHDID-2 mice (terreic acid, pergolide, and apremilast) were also effective at reducing consumption of both morphine and nicotine (26, 35, 36). It is important to note that our previous work has shown that terreic acid and pergolide did not reduce water or saccharin intake and had no effect on locomotor activity (26). Furthermore, while we have reported that iHDID-2 mice do show an apremilast-mediated reduction in water and saccharin intake, iHDID-1 mice do not (35). When administered to non-treatment-seeking humans with AUD, apremilast robustly reduced ethanol drinking, and participants continued the medication despite the adverse side effects (35). This may potentially be the case here, which would require additional investigation into the selectivity of apremilast’s effects on alcohol and intake of other abused substances. Interestingly, morphine intake was not altered when mice were treated with naltrexone, a current pharmacological intervention for OUD. Along with this, varenicline had mixed effectiveness in reducing nicotine intake, as iHDID-2 mice did not respond to treatment. It is possible that repeated administration may be required, but these findings align with the current literature in humans. Naltrexone therapy has a high relapse rate, with roughly 24% of individuals using naltrexone failing to maintain abstinence (11), and varenicline is only effective in reducing nicotine intake in 44% of individuals (12, 13). In C57BL/6J mice, naltrexone reduced ethanol intake during a DID assay (67) and oral morphine preference (68). The ineffectiveness of naltrexone on iHDID-1 and iHDID-2 mice may have a genetic component, as naltrexone did not reduce ethanol consumption in HDID-1 mice (17). This potential genetic component to naltrexone resistance may explain the high relapse rate in naltrexone therapy in humans (11). Ongoing work is being conducted to determine if treatment resistance is related to differences in genetics between iHDID-1 and iHDID-2 mice and their HS/Npt founders.

**Figure 6:**
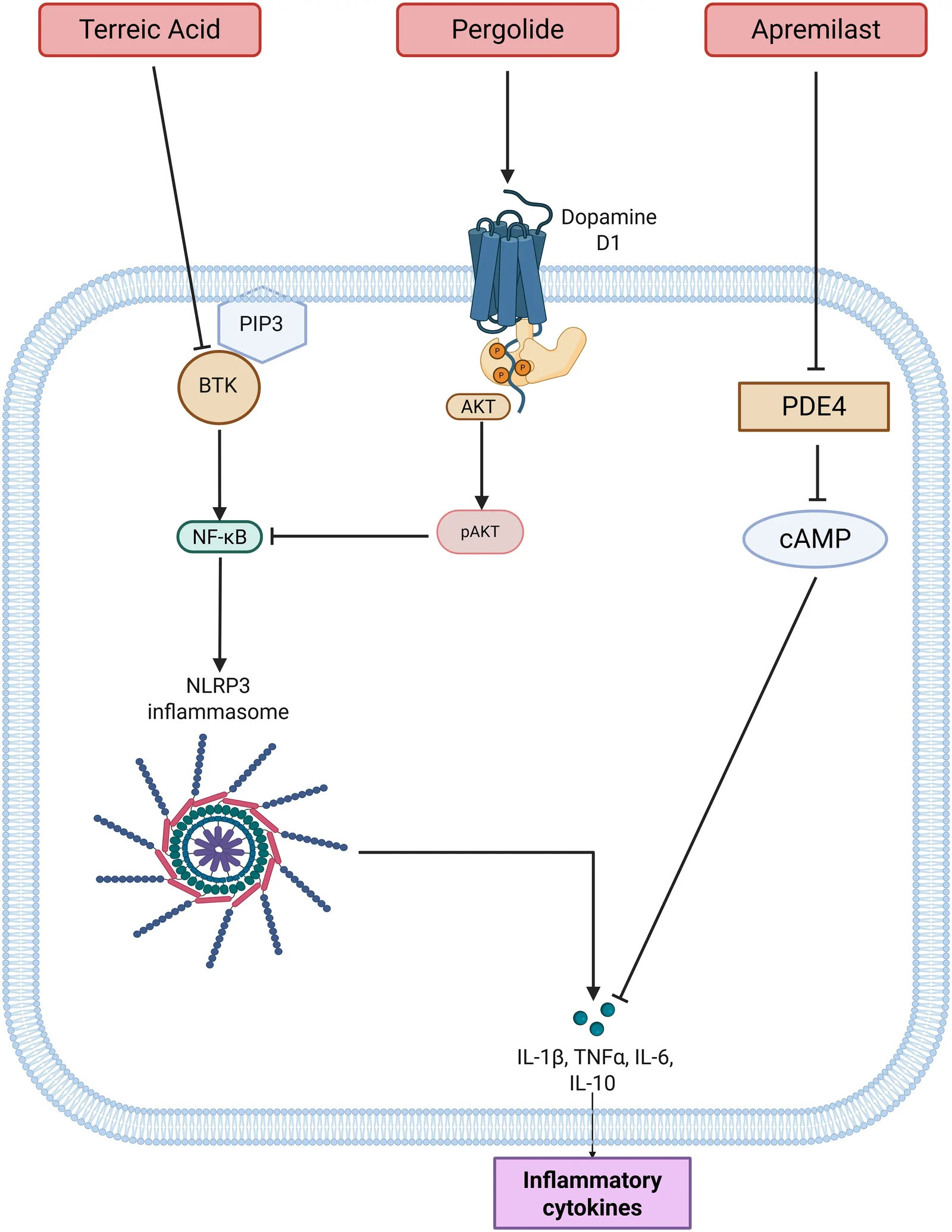
Anti-inflammatory pathways for terreic acid, pergolide, and apremilast. Inflammatory cytokine expression is reduced by terreic acid, pergolide, and apremilast through different mechanisms. These compounds all converge onto the same inflammatory markers, reducing cytokine expression. Terreic acid and pergolide reduce inflammation through manipulation of NF-κB. Apremilast reduces inflammation through increased cAMP levels. Created with Biorender.com.

As previously mentioned, morphine and nicotine increase inflammatory signaling (69–72), namely, by increasing concentrations of tumor necrosis factor α (TNF-α) (69, 71) and interleukin-10 (IL-10) (70, 72). While the primary mechanism is different, all three of the tested compounds reduce inflammation by converging mechanisms onto cytokine expression (see **Figure 6** for a simplified diagram). As a BTK inhibitor, terreic acid reduces inflammation in both the Central nervous system (CNS) and periphery (73, 74). The pro-inflammatory response to morphine and nicotine exposure may be counteracted by terreic acid, resulting in a behavioral outcome of reduced intake. This may also be the case with pergolide, which inhibits the NLRP3 pathway and reduces inflammation by way of dopamine agonism (27, 28, 75, 76). Finally, the anti-inflammatory effects of apremilast have been well documented (29–37), with PDE4 inhibitors being shown to reduce oxidative stress (77), modulate pro-inflammatory cytokines (77–79), and directly inhibit neuroinflammation by inhibiting microglia activation and restoring cAMP/PKA activity (79). Our findings here align with findings from Lai et al. (2014), where the PDE4 inhibitor rolipram attenuated heroin seeking in rats. Ongoing work includes testing these compounds as an intervention for multiple drugs of abuse and polysubstance use.

Restoration of GABA signaling may be another mechanism of apremilast that results in reduced nicotine and morphine intake. Increased GABA signaling is involved in nicotine (80, 81) and nicotine dependence (82). Furthermore, GABA neurons have been shown to express µ-opioid receptors (83), and µ-opioid receptor agonism results in reduced Ventral tegmental area (VTA) GABA neuron activity (84). One of the proposed mechanisms for apremilast-mediated reductions in ethanol consumption is through the phosphorylation of α1β3γ2-containing GABA_A_ receptors, enhancing GABAergic signaling (85). This enhancement of GABAergic signaling is present in the central amygdala (36), a region that also expresses µ-opioid receptors (86). Nicotine has been shown to reduce inhibitory signaling in the central amygdala as well (87). Correcting the altered inhibitory tone following both morphine and nicotine exposure via GABA enhancement from α1β3γ2 phosphorylation may likely result in the reduced morphine and nicotine consumption reported here.

This study highlights the use of two lines of the inbred mouse strains, iHDID-1 and iHDID-2, for the purpose of testing promising compounds in a high-throughput manner for early-stage drug use. Because these mice voluntarily consume ethanol, morphine, and nicotine, and they achieve pharmacologically relevant blood concentrations, their utility for studying polysubstance use is clear. Furthermore, we show that compounds with anti-inflammatory actions, at doses which were previously shown to selectively reduce ethanol consumption (21, 26, 35, 36), also aid in dramatically reducing morphine and nicotine intake. Finally, both the data here, from our previous work (26) and others (88–91), emphasize the use of genomics studies to discover effective therapies for substance use disorder treatments.

### Limitations and future directions

Our studies model early-stage drug use for the purpose of identifying interventions via high-throughput compound screening. As such, it is important to test our most promising translational compounds in other assays, including after chronic intake. Additionally, while the developmental timeframe of iHDID mice is beneficial for early to middle adulthood, the age range reported in these experiments could have influenced nicotine and morphine consumption in an unpredictable manner. This could be resolved with future testing across multiple developmental timepoints. With this, while nicotine and morphine intake was previously compared to HS/Npt mice (19), the findings here did not use HS/Npt for control purposes; thus, the efficacy of the compounds tested here requires future testing in a heterogeneous population. Furthermore, as both iHDID-1 and iHDID-2 mice will consume other drugs of abuse, exploring polysubstance use with iHDID mice would greatly benefit substance use research. Exploring polysubstance use with iHDID mice could be conducted with a two-bottle choice assay, offering a potentially impactful model of human polysubstance use. Additionally, continued testing in genetic animal models, such as iHDID mice, offers the benefit of strengthening the translational potential for promising compounds. For example, a recently published study implicated G-protein coupled receptor subtype 3 as a target to reduce early-stage nicotine consumption (92).

## Data Availability

The raw data supporting the conclusions of this article will be made available by the authors, without undue reservation.
